# Six-Months Retention on Treatment and Attrition Risk Factors among People Living with HIV in Kibera Informal Settlement, Nairobi, Kenya

**DOI:** 10.3390/ijerph191912657

**Published:** 2022-10-03

**Authors:** Samuel Opondo Muhula, John Gachohi, Yeri Kombe, Simon Karanja

**Affiliations:** 1 School of Public Health, Jomo Kenyatta University of Agriculture and Technology, Nairobi P.O. Box 62000-00200, Kenya; 2 Amref Health Africa, Nairobi P.O. Box 30125-00100, Kenya; 3 Kenya Medical Research Institute, Nairobi P.O. Box 54840-00200, Kenya

**Keywords:** HIV/AIDS, antiretroviral therapy, retention, attrition, Kenya

## Abstract

Early retention of patients on HIV treatment is vital in preventing new infections, reducing transmissions, preventing AIDS related deaths and achieving viral suppression. This study sought to determine the effectiveness of non-cash intervention (reminding HIV positive patients at every clinic visit that they stand to receive free T-shirts of their favorite football team or free *Kiondos* based on preference if they made it to the sixth month visit without missing a treatment appointment) and psychosocial support on retention during the first six months of HIV treatment. This unblinded randomized control trial was conducted at three health centers within the Kibera informal settlement in Nairobi, Kenya. Participants were randomly assigned to the intervention and control groups at a ratio of 1:1. Eligible participants were patients who newly tested HIV positive and enrolled for treatment at the study sites, were 18 years and older and were willing and able to provide informed consent to participate in the study. The primary outcome of interest was retention on treatment at six months. The overall retention on treatment at six months was 93%. Retention at six months among the intervention and control groups was 94% and 91%, respectively (aRR: 1.03; 95% CI: 0.98–1.09; *p*-value = 0.24). Attrition from treatment was significantly associated with being divorced, being single/never married, time to clinic, participant weight and being on other first line ART regimens other than TDF/3TC/DTG and TDF/3TC/EFV. Mortality and lost to follow-up rates were 1.6 and 13.5 per 100 person-years, respectively. The combination of non-cash incentives and psychosocial support did not improve retention during the first six months of HIV treatment. To reduce further attrition in the early stages of HIV treatment, innovative strategies are needed to reach divorced and not married/single patients earlier and support them to remain on treatment. Efforts should also be made to further decentralize ART treatment to reduce costs and time associated with travelling to and from hospitals.

## 1. Introduction

The first case of HIV in Kenya was detected in 1984 to become one of the major causes of mortality and placed tremendous strain on the health system and the economy. Towards the end of 1987, HIV began to spread rapidly and, by 1999, HIV was declared a national disaster. In 2020, Kenya had the eighth and seventh largest epidemic in the world and Africa, respectively. There were 1.4 million (1.3–1.7 million) people living with HIV (PLHIV) in the country, with an estimated prevalence of 4.2% (3.7–4.9) among adults aged 15 to 49 years. HIV is more prevalent among women than men, 870,000 (750,000–1,000,000) women aged 15 years and above live with HIV compared to 448,000 men and about 92% (79–100%) women aged 15 years and above were receiving Antiretroviral therapy (ART) compared to 84% (74–98%) of men [1,2]. More women compared to men are on HIV treatment.

Early in the HIV/AIDS epidemic, there were few targets to guide the global response to the HIV/AIDS pandemic. To date, these have increasingly developed into numerous and complex target frameworks. The first global HIV/AIDS targets were first defined in the 2000 millennium development goals (MDGs). Though there were no specific quantitative targets, the MDGs stated an aim to ‘halt and reverse’ the epidemic [3]. In 2001, there was “The Declaration on Commitment” on HIV/AIDS which set out more timebound goals and targets, placing importance on transparency, accountability and ongoing reporting for concerted, determined civil society activism [4]. Progress towards the 2001 Declaration’s targets was reported in 2006, indicating underperformance on most of the targets apart from that of increasing HIV/AIDS funding for low-and-middle-income countries (LMICs), which had been achieved [5]. In the 2011–2015 strategy, Joint United Nations Programme on HIV/AIDS (UNAIDS) set a global vision to achieve Three Zeros—zero new HIV infections, zero AIDS related deaths and zero discrimination [6]. Five years later, in 2015, the Three Zeros served as the basis for the development of the fast-track strategy, outlining 10 targets, one of which was the 90-90-90 target for 2020, which served as a very important pointer of progress for countries [7]. These targets were missed, but not by a big margin. At the end of 2020, 84% of people living with HIV (PLHIV) knew their HIV status, 87% of PLHIV who knew their HIV status were accessing ART, and 90% of people under treatment were virally suppressed [8]. Kenya’s performance against the first 90 improved from 71% in 2010 to 96% in 2020, while performance against the second 90 improved from 44% in 2010 to 89% in 2020, and performance against the third 90 was at 94% in 2020, well above the target of 90%. Generally, Kenya’s performance as of 2020 seemed encouraging across all the HIV testing and treatment cascade, but a cursory look at the absolute figures show about 19,000 AIDS related deaths in 2020, 33,000 adults and children newly infected with HIV in the same year, and an estimated 350,000 PLHIV who are not yet on HIV treatment [1,2]. These gaps are even larger within sub-populations, including children, young people and men. The annual number of AIDS-related deaths and new HIV infections in Kenya has hardly changed over the last five years, far short of the UN General Assembly targets set in 2016. The fast-track targets have since been revised to 95-95-95 by 2030 [9].

Although a great deal of research has been done on the long-term retention of patients on treatment programs in sub-Saharan Africa (SSA), early retention of patients on treatment has received comparatively less attention. Evidence from various studies have shown that ART is effective in preventing progression to AIDS and HIV transmission if treatment is started immediately [10,11]. But not all patients remain on treatment under programmatic conditions. Some patients drop out of treatment and end up not restarting treatment elsewhere. Such patients are at high risk of morbidity and death within a short time. 

There is limited evidence from resource-constrained settings and SSA on interventions and strategies targeted at improving early retention of patients on treatment [12,13]. It is depressing and even scandalous that after more than thirty years into the global HIV epidemic, effective methods to support treatment efforts remain limited to biomedical interventions and besides, researchers have conducted very few rigorous evaluations of such interventions [14,15].

To address the research gaps, this randomized control trial (RCT) tested a novel interventions of non-cash incentive where participants in the treatment arm of the study received a reminder at every clinic visit that they stand to receive free T-shirts of their favorite football team or free *Kiondos* based on choice if they made it to the sixth month visit without missing appointment and were enrolled in and attended all (monthly) psychosocial support groups for the six months. The study was implemented in Kenya, within the Kibera informal settlement in Nairobi with the focus on retaining patients undergoing HIV treatment for the following reasons. First, the third goal of the Sustainable Development Goals (SDGs) set by the United Nations is to ensure healthy lives and to promote well-being for all at all ages. This research has the potential to contribute to this goal by expediting Kenya’s progress towards the achievement of UNAIDS second and third Fast-Track targets by the year 2030. Second, if the intervention is effective in reducing patient attrition from treatment, then the research would contribute in reducing morbidity and mortality among PLHIV, which currently stand at an average of 20,374 AIDS related deaths recorded in the year 2020 [1,2]. Third, early initiation of PLHIV on ART, retaining them on ART and ensuring virological suppression has significant clinical benefits for treatment and can eliminate HIV transmission [16]. Several novel approaches that address structural and behavioral risk factors should be considered at local levels for maximum impact on the epidemic [17]

The broad objective of this study was to determine the effectiveness of non-cash incentives combined with psychosocial support on HIV treatment retention in the first six months of ART among patients in the informal settlement of Kibera in Nairobi, Kenya. The specific study objectives were: (1) To determine the effectiveness of the intervention on retention of patients on HIV treatment in the first six months of starting ART, (2) To determine patient level factors associated with attrition of patients from HIV treatment, and (3) To determine survival estimates of patients on HIV treatment.

## 2. Materials and Methods

### 2.1. Study Sites and Area

The study participants were recruited from three health facilities of Kibera Community Health center, Kibera South Health Center and Silanga Dispensary, all situated within Kibera Informal Settlement, where the majority of the participants reside. The Kibera informal settlement is located 5 km southwest of Nairobi Central Business District. It is made up of 14 villages with about 90 health facilities [18]. Over the years, Kibera has grown to be a cosmopolitan society comprising major ethnic communities in Kenya, and incorporating the two major religious groups in Kenya: Christians and Muslims [19]. It is one of the largest urban informal settlements in Africa, with an HIV prevalence of 12.6% compared to the country’s HIV prevalence of 4.9% [20], and a 12-month patient attrition from HIV care of 19% [21]. Residents in Kibera are highly mobile to and from upcountry, live in inhumane conditions with a lack of clean water, housing, health services and a lack of solid waste management facilities. In addition, Kibera slum dwellers face inadequate schooling facilities, unemployment, high crime rates, and insecurity, which has led to mass poverty, conflicts, contagious diseases and other social, ecological and economic risks [19]. Participant recruitments at Kibera Community Health Center started on 4 February 2019 while recruitment at Kibera South Dispensary and Silanga Dispensary started on 1 April 2019.

### 2.2. Study Participants

Study participants included individuals who had recently tested HIV positive at the study sites, who were then referred to a study peer educator/community health volunteer (CHV) for assessment of study eligibility and consenting. Inclusion criteria were patients who had recently tested HIV positive, were 18 years and older, and were able and willing to provide informed consent to participate in the study. Exclusion criteria were those patients who previously enrolled in ART either at the study site or at any other health facility.

### 2.3. Study Design

The study adopted an experimental design: a randomized control trial with control and intervention groups. Group 1 received the intervention in addition to the standard of care, while the control (group 2), received the standard of care only.

### 2.4. Study Procedures

A total of 10 peer educators and a team of health facility staff at the three study sites were trained on the study procedures and the general details of the study protocol. These included the process of identification and consenting the participants, how to complete the baseline questionnaire using the handheld tablets and the study randomization process. The peer educators connected with newly diagnosed HIV positive patients to escort them to care and treatment service points and linked them to appropriate psychosocial support groups. The same peer educators traced the participants whenever they missed clinic appointments and made home visits where necessary.

Initially, the intervention group was to receive the following combination of interventions: (1) a thank you note issued at every clinic visit, thanking the participants for attending clinic appointments and a providing them with a reminder of the next appointment date; (2) a reminder at every clinic visit that they could receive free T-shirts of their favorite football team or free *Kiondos* based on choice; (3) a certificate of appreciation if they made it to the six month visit without missing an appointment and (4) enrollment into psycho-social support groups. To inform the choice of the intervention and specifically the incentives, the principal investigator (PI) visited one psychosocial support group and sought their opinion on the proposed incentives. The majority were happy with the proposal to receive free T-shirts of their favorite football team or a free *Kiondo* but were completely unreceptive of the proposal to issue them with small thank you notes at every facility visit. They suggested that the card would not be useful and that the clinicians could still appreciate them by word of mouth. They were also not receptive of the certificates of appreciation at the end of the sixth month visit. This therefore reduced the intervention to a combination of: (1) a reminder at every clinic visit that the participants stand to receive free T-shirts of their favorite football team or free *Kiondos* based on choice if they made it to the six months visit without missing appointments; and (2) enrollment into psychosocial support groups of which meetings were held once a month.

The recruitment of participants into the study involved an eligibility assessment and consent at the three study sites. The officers at the clinics introduced the patients to the peer educators upon their expression of interest in the study. The peer educators then identified a quiet and private place and provided a detailed explanation of the study and sought informed consent. The peer educators then took the consented participants through baseline study procedures, which included completing the baseline questionnaire.

### 2.5. The Intervention

Participants were randomly assigned to receive the intervention in addition to the standard of care or standard of care only. The intervention consisted of a reminder at every clinic visit that the participants stand to receive free T-shirts of their favorite football team or free *Kiondos* based on preference if they made it to the sixth month visit without missing a treatment appointment and attending all psychosocial support group meetings. Not missing a treatment appointment meant that the participant attended all clinic visits one week before or after the appointment dates. The psychosocial support group meetings were convened once a month on a weekend and structured in two parts. Part one took about an hour and involved experience sharing on how one was doing and health education on various topics such as self-acceptance, nutrition, adherence, disclosure, hygiene, opportunistic infections, self-stigma, condom use, treatment failure, positive living and how to cope with HIV/AIDS. The second part of the meetings ran for about 30 min and involved discussions on how to improve participants’ economic status through involvement in various income generating activities (IGAs). The meetings were held within the health facility compound. Tea and snacks were provided. The choice of these interventions was informed by the fact that majority of people in Kenya are emotionally attached to English and local football premier leagues and this project intended to leverage the attachment to football to keep patients on HIV treatment. In addition, Kenya banned plastic bags in 2017, and so *Kiondos* would be preferred by the participants as a durable shopping bag. The choice of psychosocial support groups was informed by a preliminary project that Amref Health Africa was implementing in two health facilities within the informal settlement of Kibera. In the project, patients were enrolled in the groups, and they had received and attended the psychosocial support groups, hence providing evidence that these were promising interventions.

**Figure 1:**
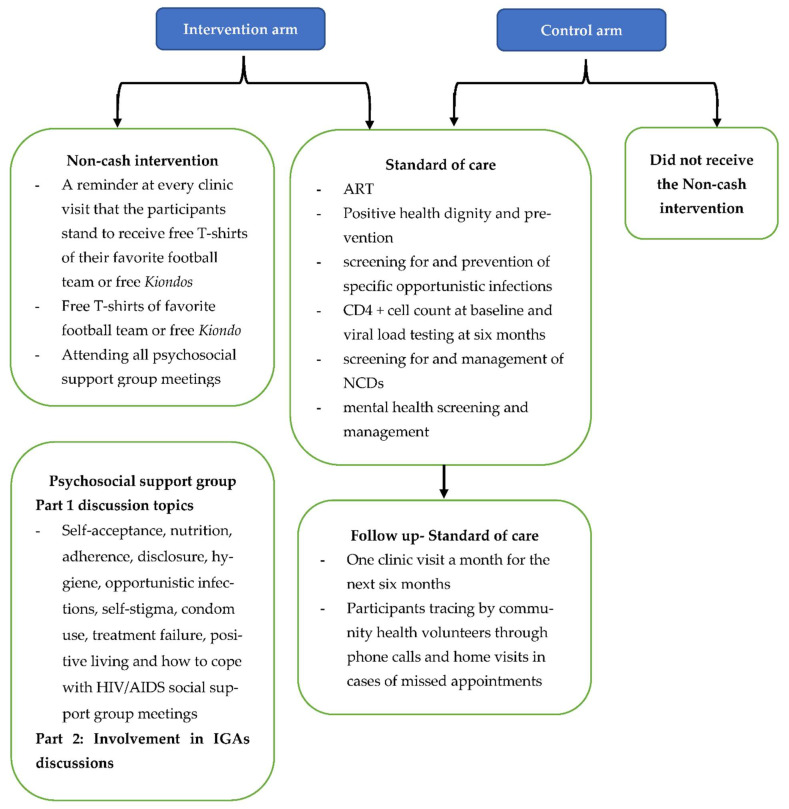
Intervention and control study arms.

Both the intervention and control groups received the standard care as defined in the ministry of health test & treat guidelines [22]. These included the following seven components: (i) ART; (ii) positive health, dignity and prevention; (iii) screening for and prevention of specific opportunistic infections; (iv) CD4 + cell count at baseline and viral load testing at six months; (v) screening for and management of non-communicable diseases; (vi) mental health screening and management; and (vii) participant tracing by peer educators through phone calls and home visits in cases of missed appointments. Patients who did not attend a clinic appointment were called one day after a missed appointment, a second time three days later and a final time seven days later, after which the peer educators could make a home visit to find out why the client had not attended clinic appointment. See Figure 1 below, an illustration of the intervention and control arms.

### 2.6. Randomization and Allocation of Study Participants

Participants were randomly assigned to the intervention and control groups at a ratio of 1:1. Randomization assignments were provided in opaque sealed envelopes, and after consenting, the participants were asked to select one envelope and to break the seal to reveal the randomization assignment group. Both the participants and the study team were blinded to the intervention assignment for logistical purposes.

Concerns of patients in the control arm feeling disenfranchised and being transferred out to other health facilities if they realized that patients on the intervention arm of the study were receiving incentives could have led to high dropout rates. This was, however, mitigated by clearly informing the participants of the study procedures and involving them in the randomization process where they selected one randomization envelope and revealed the assignment. The other concern was a possibility of spillover of the information gained from the psychosocial support groups to those in the control group. This was addressed by ensuring that patients in the control group were scheduled for appointment visits on separate days from those in the treatment group.

### 2.7. Data Collection

Peer educators conducted participants’ eligibility screening assessment, looking at inclusion, exclusion criteria and obtained consent for study enrolment. If a patient was eligible and willing to participate in the study, the peer educator went through the consent form with the participant and collected either written consent for the literate participants or a thumbprint with a witness signature if illiterate. The peer educators then administered a piloted, validated questionnaire programmed in Open Data Kit (ODK) to capture participants’ socio-demographic characteristics, HIV care, social life and labor market involvement data. The participants had the choice of conversing in English or Kiswahili.

A health facility data extraction tool was used to extract additional data from health facility electronic medical records systems. The data elements extracted were patient identification number, health facility where first positive HIV test was done, date confirmed HIV positive, ART start date, CD4 count, viral load count, WHO staging, Tuberculosis (TB) screening outcome, patient outcome, exit reason and exit date.

At six months, study specific follow-up questionnaires (electronic) were administered to all participants who returned to the clinic. At the end of the study, a tracing report form was filled out for all participants, indicating whether they returned to the clinic or if they did not, what type of tracing was undertaken to ascertain their status (telephone or home visits). This form captured the participants’ final outcome i.e. retained in clinic, transferred out, died or lost to follow-up (LTFU).

### 2.8. Ethical Considerations

The study protocol was reviewed and approved at the Amref Health Africa in Kenya Ethics and Scientific Review Committee before starting data collection. Ethical approval was renewed annually to allow for the completion of data collection. The approval was granted on 3 December 2018 with a reference number: AMREF-ESRC P550/2018.

Informed consent was sought from all study participants in the language in which they were most comfortable in, English or Kiswahili, including being informed about the study risks and benefits and measures in place to ensure anonymity and confidentiality of the data collected. Each participant was given the opportunity to ask questions before providing written consent. Once signed, each participant was provided with a copy of the informed consent form. Illiterate participants provided consent in the presence of a literate witness and provided thumbprints instead of signatures.

All data obtained from electronic medical records systems were stripped of personal identifiers and assigned unique identifiers. Under no circumstances has any identifying information on individual participants been made public.

### 2.9. Outcomes

The primary outcome was retention on HIV treatment at six months, defined as the proportion of participants retained on treatment at six months from treatment enrollment and measured by whether the participant attended a follow-up appointment within the 5 to 7-month timeframe from treatment enrollment. Participants confirmed to be active on treatment at some other health facilities were considered retained on treatment. Participants not reachable over the phone were physically traced within the community by peer educators to ascertain the final status. Those not traced through phone calls or physically were considered LTFU.

The secondary outcomes were viral suppression, labour market participation, HIV disclosure, sexual behavior and survival on treatment.

### 2.10. Statistical Analysis

A data analysis was performed according to the intention-to-treat (ITT) principle where all study participants were analyzed according to their initially assigned study arms at baseline, regardless of adherence to study protocol. Participants who died, were LTFU, were confirmed as retained at the clinic of recruitment and those confirmed to have transferred to other clinics and continued to be on ART were included in the ITT analysis.

Baseline characteristics were reported per randomization arm to enable comparison of groups. Dichotomous variables were summarised as a proportion of patients with the count divided by the total number of evaluated patients. Continuous variables were summarised as mean with standard deviation (SD) in case of normal distribution and as median with interquartile range (IQR) in case of non-normal distribution. For continuous variables, a footnote stating the number of evaluated participants was included.

Balance in baseline variables across the two groups was assessed by calculating the standardized difference in baseline variables between the two groups using Cohen’s d and the difference in means or proportions divided by the pooled standard deviation. An absolute standardized difference >0.10 was evidence of imbalance worth noting and interpreted within the clinical context and the strength of the variable’s relationship with the outcome [23].

The primary outcome, six-month retention on HIV treatment, was assessed using adjusted risk ratios (aRR), with the differences in proportions among the treatment and control group presented with a 95% confidence interval (CI) along with a *p*-value associated with the aRR. Secondary outcomes were similarly assessed and presented with a 95% CI for the differences in the two groups. Adjusted hazard ratios (aHR), 95% CI and *p*-values were calculated to estimate the association between attrition and variables at an individual level. For time-to-event outcomes, i.e., the effect of the interventions on the time to retention (remaining on treatment after ART initiation i.e. the time between study enrollment and the last scheduled visit) was examined with a Kaplan-Meier plot; equality of survival functions was tested using the log-rank test. The individual factors associated with attrition from treatment were examined using a Cox proportional hazards model and presented hazard ratios for each time interval and 95% CIs. Mortality and LTFU rates were calculated per 100 person-years.

## 3. Results

### 3.1. Socio Demographic and Clinical Characteristics of Study Participants

The mean age of the study population was 34 years (SD 9.9), and 57% were male. The majority (45%) were aged between 25–34 years, followed by those aged between 35–44 years at 25%. Almost all (98%) of the participants were Christians and a majority (60%) were married to either one or more than one partner. The majority (96%) had at one time or another attended school and 52% completed primary school, 15% completed secondary school and 4% completed tertiary school. One-third (30%) of the study participants did not have formal education. Twenty percent (20%), twenty three percent (23%), thirty five percent (35%), sixteen percent (16%) and six percent (6%) of the households were made up of one, two, three, four and more than five people, respectively. The majority (89%) were residents within the Kibera informal settlement. Ninety percent (90%) walked to the clinics as a mode of transport, spending a medium time of 30 min (IQR 15–30) to access health services. Unemployment rates among study participants were high, with 64% and 36% having formal employment. All (99%) of the study participants were HIV asymptomatic, i.e., WHO HIV Clinical stage 1 and either received first line ART fixed-dose combination of Dolutegravir/lamivudine/tenofovir (DTG/3TC/TDF- 51%) or Tenofovir/lamivudine/Efavirenz (TDF/3TC/EFV-48%). The median CD4+ count was 394.5 cells/ul (IQR 172–639), while the mean weight was 64.6 kg (SD 19.7) with 47% of the participants’ weights documented. Participant characteristics between the two groups were balanced, with the exception of the predominant mode of transport to clinic (d = −0.17), occupation (d = 0.12), weight (d = −0.15) and WHO HIV Clinical staging (d = −0.22). See Table 1 below

### 3.2. Retained on Treatment at Six Months

**Figure 2:**
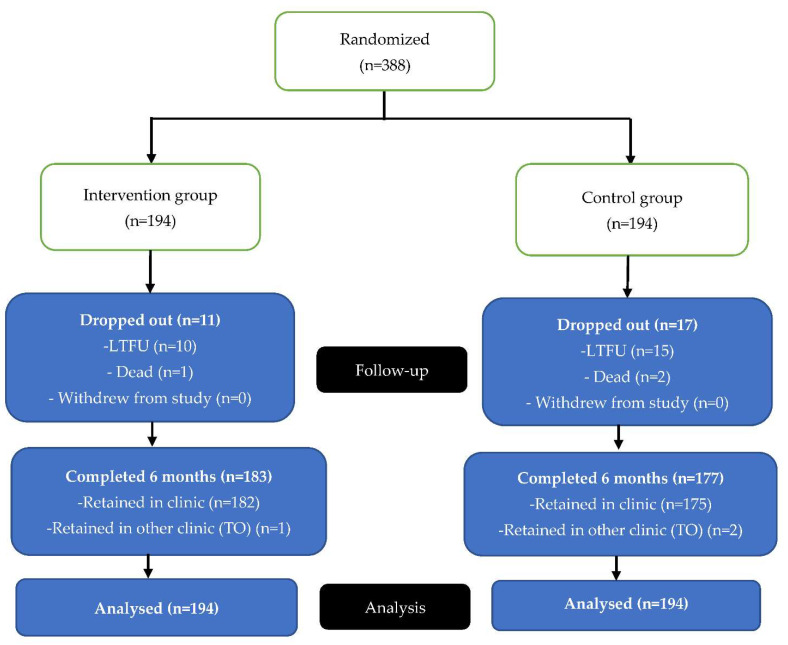
Study participants as included in the analysis.

A total of 388 study participants were randomly assigned to the intervention and control arms and were followed up for an average period of six months. Of these, 360 (93%) were retained on treatment including three (3) participants who transferred out to other health facilities and continued to receive treatment. Among the 28 (7%) participants not on treatment, three (3) had died and 25 could not be traced after exhaustive tracing attempts (lost to follow-up). See Figure 2 below.

Retention on treatment at six months among the intervention and control groups was 94% (95% CI: 90–97%) and 91% (95% CI: 86–95%), respectively. The risk of the participants being retained on treatment was 1.03 times as high in the intervention group compared to the control group over a six-month period (aRR: 1.03; 95% CI: 0.98–1.09; *p*-value = 0.24).

The viral suppression rate at six months was 93% (95% CI: 87–96%) among the intervention group, compared to 97% (95% CI: 92–99%) among the control group. The risk of viral suppression was 0.96 times as high in the intervention group compared to the control group over a six-month period (aRR: 0.96, 95% CI: 0.91–1.01; *p*-value = 0.12). The overall VL suppression rate at six months was 95% (95% CI: 91–97%).

Labor market participation rates for the intervention and control groups were 40% (95% CI: 32–49%) and 43% (95% CI: 35–52%), respectively. The risk of missing work for a whole day or more was 0.93 times as high in the intervention group compared to the control group over a six-month period (aRR: 0.93, 95% CI: 0.71–1.24; *p*-value = 0.63). The overall labor market participation rate was 42% (95% CI: 36–48%).

HIV status disclosure rates were 71% (95% CI: 64–77%) and 73% (95% CI: 66–79%) among the intervention and control groups, respectively. The risk of participants disclosing their HIV status was 0.97 times as high in the intervention group compared to the control group over a six-month period (aRR: 0.97, 95% CI: 0.86–1.10; *p*-value= 0.65). The overall HIV status disclosure rate was 72% (95% CI: 67–76%).

The reported condom-use rates the last time a participant had sex with an unofficial partner were 79% (95% CI: 66–88%) and 82% (95% CI: 69–90%) among the intervention and control groups, respectively. The risk of using a condom with an unofficial partner was 0.97 times as high in the intervention group compared to the control group over a six-month period (aRR: 0.97, 95% CI: 0.81 to 1.17; *p*-value = 0.77). The overall reported condom use rate was 80% (95% CI: 72–87%). See Table 2 below.

### 3.3. Factors Associated with Attrition

Table 3 below shows the final multivariate model and the adjusted hazard ratios (aHR) for each of the factors associated with attrition at *p*-value < 0.05. The risk factors were being divorced (aHR: 631,925.9, 95% CI: 4560.265–8.76 × 10^7^; *p*-value < 0.000), never married/single (aHR: 7.83, 95% CI: 2.02–30.34; *p*-value = 0.003), time to clinic (minutes) (aHR: 2.86, 95% CI: 2.62–3.13; *p*-value < 0.000), participant weight (aHR: 1.64, 95% CI: 1.55–1.74; *p*-value < 0.000) and being on other 1st line ART regimens other than TDF/3TC/DTG and TDF/3TC/EFV (aHR: 1.42 × 10^21^; 95% CI: 1.92 × 10^20^–1.05 × 10^22^; *p*-value < 0.000).

### 3.4. Survival in HIV Treatment

**Figure 3:**
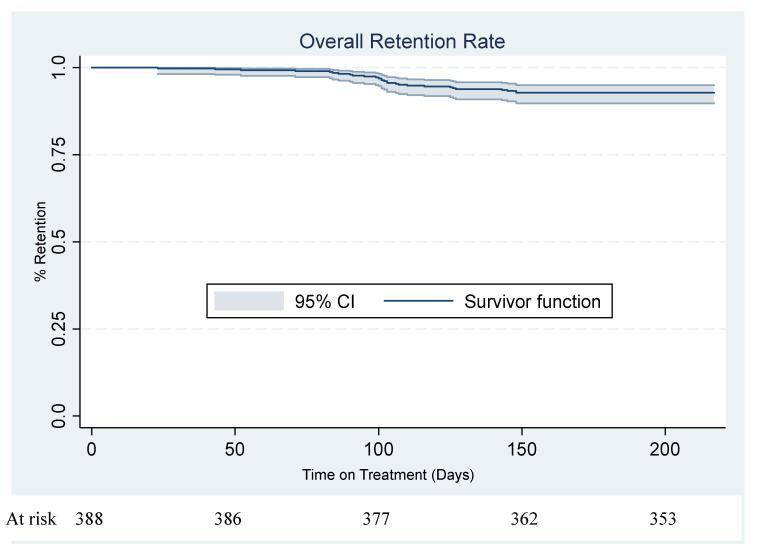
Kaplan-Meier overall retention rate.

**Figure 4:**
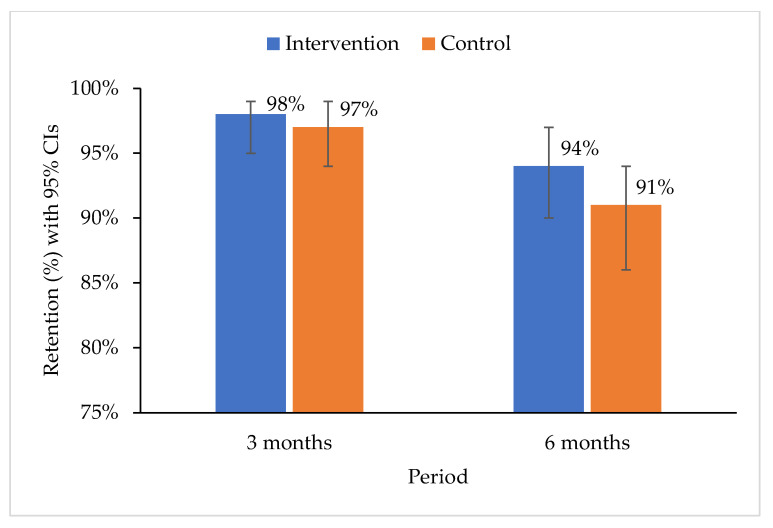
Retention rates for intervention and control groups at three and six months.

**Figure 5:**
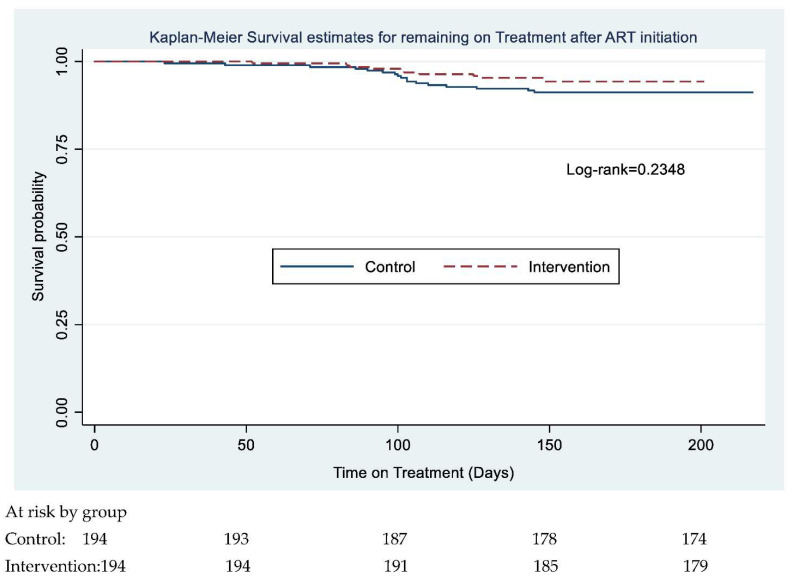
Kaplan-Meier survival estimate for remaining on treatment after ART initiation.

The overall retention on treatment at three and six months were 98% (95% CI: 96–99%) and 93% (95% CI: 90–95%) respectively (Figure 3). The overall attrition rate was 14.7 drop-outs per 100 person-years among the 388 participants. This meant that there will be on average 14.7 drop-outs if 100 patients were followed-up for one year. The mortality rate was 1.6 per 100 person-years, and the LTFU rate was 13.5 per 100 person-years. The retention rate among the control and intervention groups was 97% (95% CI: 94–99%) and 98% (95% CI: 95–99%) at three months and 91% (95% CI: 86–95%) and 94% (95% CI: 90–97%) at six months (Figure 4). A test of equality of the two groups using a log-rank test indicated that the survival functions for the treatment and control groups were equal (ꭓ^2^(1) = 1.41, *p*-value = 0.2348) (Figure 5).

## 4. Discussion

This study evaluated whether promising patients newly initiated on ART stayed in treatment for six months, after which they would be provided with free T-shirts of their favorite football team or free *Kiondo* on condition that they attended all psychosocial support group meetings. The intervention was evaluated as to whether it would improve retention on HIV/AIDS treatment among resource limited populations in the Kibera informal settlement in Nairobi, Kenya. The finding was that the intervention was not effective in retaining the patients on ART during the first six months of treatment. Even though the intervention was not effective, the retention rate of 93% at six-months was high and consistent with data from a study conducted in Tanzania which documented the six-month retention rate at 93.5% [24]. This was much higher than data documented from studies conducted in Kenya, including one conducted within the same locality of the Kibera informal settlement [25,26,27]. The attrition rate among the 388 patients on ART was 14.7 per 100 person-years, much better than the 23 per 100 person-years that was documented at a rural HIV/AIDS clinic in Coastal Kenya and within the same locality of the Kibera informal settlement [26,27]. Mortality and LTFU incidence rates were 1.6 and 13.5 per 100 person-years, respectively.

The overall six-month retention on treatment in this study population was 93%, which was not any different from retention in the clinics (92%), given that only three participants were confirmed to have transferred to other health facilities and continued to receive treatment. Similarly, retention on treatment was not any different among adults randomized to the intervention (94%) compared with the control (91%). It is, however important to consider clients who are active in treatment in other health facilities when quantifying retention on treatment, hence the need to put in place adequate structures for tracing patients who miss clinic appointments. Of note, all participants who could not be traced for a period of three months (90 days) were subsequently documented as LTFU from HIV/AIDS treatment.

Attrition from HIV treatment depended on being divorced, not married/single, time to clinic, patient weight and being on other first line ART regimens other than fixed-dose combinations of tenofovir/lamivudine/dolutegravir (TDF/3TC/DTG) and tenofovir/lamivudine/efavirenz (TDF/3TC/EFV). Patients who were divorced or not married/single were at higher risk of attrition from HIV treatment compared to those who were married, as they may lack the needed social and psychological support to adequately deal with the stigma and overall economic and social burden associated with HIV infection. They were therefore more prone to attrition from HIV treatment, which is consistent with previous work done in Kenya, South Africa and India [28,29,30]. Patients with higher weights had a higher probability of being retained on treatment compared to those with lesser weights, consistent with findings from studies conducted in Haiti and Kinshasa in the Democratic Republic of Congo [31,32]. In accordance with studies in Kenya and India, patients with longer travel times to clinic were at a higher risk of attrition from treatment, as they may lack the motivation to walk long distances to the clinics or may not have the money to pay fares to and from the clinics [30,33], which support the early initiative by the Ministry of Health to decentralize ART treatment sites to lower level health centers and dispensaries to reduce time and costs associated with travelling to and from hospitals [34].

The choice of the primary outcome, early retention on treatment, underscores its vitality in reducing transmissions, morbidity and mortality, preventing new infections and achieving viral suppression [35,36]. Although retention on treatment has been explored in studies of adults in HIV services, very few have measured early (at six months from start of ART) retention on HIV treatment programs [12,13]. To our knowledge, no study in Kenya of an RCT design has explored strategies to improve early retention on treatment among adult HIV positive clients.

It is part of the standard practice for health facilities to trace participants who miss clinic appointments and therefore this was done by all the clinics involved in the study in addition to offering other support services that might have helped to engage and retain participants in treatment. This could be one of the reasons why retention among the intervention and control groups remained high with no clear effect of the intervention felt. It could also be that a reward that is not given until six months of treatment has been completed does not have much impact on behavior in the early months of HIV treatment.

The study hypothesized that by combining the interventions, we could target multiple barriers to engagement along the HIV treatment cascade challenges affecting the early retention of patients in treatment, especially those residing in slum areas. Our retention (94%) of participants in treatment at six months in the intervention arm is within the range of studies that have evaluated the effect of single interventions at six months in SSA [12]. Retention being a predictor of viral suppression, an achievement of 94% could be an indication that Kenya is well on course to the UNAIDS target of achieving viral suppression for 95% of those in treatment by 2030.

This trial advances knowledge gained from previous studies in several important ways. To the best of our knowledge, this was the first study in a resource-limited setting to test whether an incentive of this nature (free T-shirts of a favorite football team or a free *Kiondo*) combined with psychosocial support improved retention in a general population with HIV. This study was designed to evaluate a relatively simple implementation model that is feasible to administer in real-life clinical settings. The conditions for providing the incentives were relaxed provided that the patients attended clinic regardless of timeliness in order to further simplify implementation and to avoid excluding disadvantaged participants facing the greatest obstacles to keeping appointments. The focus on this group was also driven by the increased clinical, social and economic vulnerability of patients at this time and the potential to influence the formation of habits early in treatment for long lasting effects [37,38]. The findings suggest that non-cash incentives offered to adults initiating ART were ineffective at least in the short-term, though more work is needed to understand the long-term effects. Other key strengths of this study are its high participation rate and low deaths, which minimized the possibility of non-participation bias. Only 2% of those assessed for eligibility declined to participate in the trial, less than 1% of the participants died and none withdrew from the trial. By tracing participants who did not return to the clinic and categorizing those confirmed to be receiving care in other health facilities as transfer outs was a more valid assessment of retention in treatment than solely using retention in clinics of study as a proxy measure for retention on treatment. The randomized design, inclusion of a comparison group and the focus on ART initiates eliminated the pathway to presentation to care with advanced HIV/AIDS.

This study had important limitations. First, some data were missing from the electronic medical records system. However, these were not differential by study arm and would not likely result in the underestimation of retention in treatment. Some known risk factors to attrition such as a history of TB and immunosuppression could not be assessed due to the unavailability of some data such as TB status, CD4+ counts, and weights. Second, the study clinics already had mechanisms in place to support patient retention on treatment, such as calling patients who missed appointments, however these are not optimally implemented due to inadequate resources. The intervention might have been more likely to show an effect on retention on HIV treatment and other secondary outcomes if tested in settings without these support mechanisms. A small number of participants could not be located to confirm the final outcome after exhaustive tracing and were therefore classified as lost to follow-up. Although this study followed gold standard Ministry of Health and PEPFAR indicator guidelines, misclassification might have occurred if patients did not have a documented facility transfer. The proportion of untraceable participants among those not retained on treatment did not significantly vary by study arms, suggesting that potential misclassification might be non-differential by study arm.

## 5. Conclusions

The findings demonstrate that the combination of non-cash incentives of a reminder at every clinic visit that participants will receive free T-shirts of their favorite football team or free *Kiondos* based on preference if they made it to the sixth month visit without missing a treatment appointment and psychosocial support did not improve retention during the first six months of HIV treatment. However, the study confirms the high retention rates at six months of HIV treatment as documented by other studies and the interdependence between retention and viral suppression, hence the need to examine the two together to achieve the third 95% UNAIDS target by 2030. To reduce further attrition in the early stages of HIV treatment, strategies should be piloted to reach divorced and not married/single patients earlier and support them to remain on treatment. Efforts should also be made to further decentralize ART treatment to reduce costs and time associated with travelling to and from hospitals.

Although this combination intervention was not effective in improving retention in HIV treatment, its simplicity in implementation warrants serious consideration along with other proven interventions as part of a comprehensive package of support at the time of treatment initiation. New interventions to improve early retention in HIV treatment in general HIV populations are required in order to fast track the achievement of 95-95-95 UNAIDS targets by 2030. This study provides an important contribution to understanding the potential of non-cash incentives combined with psychosocial support to achieving epidemic control in resource limited settings and therefore further research investigating the long-term effects, cost-effectiveness, scalability and sustainability of such interventions are warranted.

## Figures and Tables

**Table 1:** Baseline characteristics of study participants.

Variable	Intervention Group (*n* = 194)	Control Group (*n* = 194)	Total (*n* = 388)	Standardized Difference
Sex (Male = 1)				−0.05
Male	108 (56%)	113 (58%)	221 (57%)	
Female	86 (44%)	81 (42%)	167 (43%)	
Age (Years)				
Mean (SD)	33.5 (9.4)	34.6 (10.3)	34.0 (9.9) ⁑	0.10
18–24	30 (15%)	27 (14%)	57 (15%)	
25–34	84 (43%)	89 (46%)	173 (45%)	
35–44	55 (28%)	41 (21%)	96 (25%)	
>45	25 (13%)	37 (19%)	62 (16%)	
Religion				0.00
Christian	190 (98%)	190 (98%)	380 (98%)	
Muslim	4 (2%)	4 (2%)	8 (2%)	
Marital status				−0.10
Married	109 (56%)	123 (63%)	232 (60%)	
Divorced	37 (19%)	27 (14%)	64 (16%)	
Never married/Single	23 (12%)	26 (13%)	49 (13%)	
Widowed	25 (13%)	18 (9%)	43 (11%)	
Ever attended school				−0.05
Yes	186 (96%)	188 (97%)	374 (96%)	
No	8 (4%)	6 (3%)	14 (4%)	
Education completed				0.04
No formal education	56 (29%)	59 (30%)	115 (30%)	
Primary completed	104 (54%)	96 (49%)	200 (52%)	
Secondary completed	29 (15%)	30 (15%)	59 (15%)	
Tertiary completed	5 (3%)	9 (5%)	14 (4%)	
House hold size				−0.06
One person	43 (22%)	36 (19%)	79 (20%)	
Two people	39 (20%)	50 (26%)	89 (23%)	
Three people	65 (34%)	69 (36%)	134 (35%)	
Four people	35 (18%)	26 (13%)	61 (16%)	
Five and above people	12 (6%)	13 (7%)	25 (6%)	
Financial support to				−0.07
One person	13 (7%)	12 (6%)	25 (6%)	
Two people	9 (5%)	9 (5%)	18 (5%)	
Three people	50 (26%)	50 (26%)	100 (26%)	
Four people	53 (27%)	61 (31%)	114 (29%)	
Five and above	70 (36%)	60 (31%)	130 (34%)	
Clinic				0.04
Kibera Community Health Center	110 (57%)	106 (55%)	216 (56%)	
Kibera South Health Center	45 (23%)	46 (24%)	91 (23%)	
Silanga dispensary	39 (20%)	42 (22%)	81 (21%)	
Predominant mode of transport to clinic				−0.17 *
Walking	169 (87%)	180 (93%)	349 (90%)	
Matatu	24 (12%)	13 (7%)	37 (10%)	
Boda Boda	2 (1%)	1 (1%)	2 (1%)	
Residence Status				−0.09
Kibera	169 (87%)	175 (90%)	344 (89%)	
Outside Kibera	25 (13%)	19 (10%)	44 (11%)	
Occupation				0.12 *
Salaried employment	75 (39%)	66 (34%)	141 (36%)	
Self employed/Business person	37 (19%)	35 (18%)	72 (19%)	
Casual Labour	46 (24%)	49 (25%)	95 (24%)	
Unemployed	36 (19%)	44 (23%)	80 (21%)	
Time to Clinic (Minutes)				0.01
Median (IQR)	30 (15)	30 (15)	30 (15) ‡	
CD4+ Cells per ul				0.04
Median (IQR)	394.5 (467)	395.5 (506)	394.5 (467) †	
Weight (Kg)				−0.15 *
Mean (SD)	65.9 (20.9)	63 (18.2)	64.6 (19.7) ǂ	
WHO HIV Clinical staging				−0.22 *
Stage 1	179 (97%)	181 (100%)	360 (99%)	
Stage 2	4 (2%)	0	4 (1%)	
Stage 3	1 (1%)	0	1 (0.3%)	
1st Line ART original regimen				0.03
TDF/3TC/DTG	101 (52%)	93 (49%)	194 (51%)	
TDF/3TC/EFV	87 (45%)	96 (50%)	183 (48%)	
Other ^+^	5 (3%)	2 (1%)	7 (2%)	

Data are *n* (%), mean (SD = standard deviation) or median (IQR = interquartile range). Married includes monogamous and polygamous. ^+^ Other = ABC/3TC/EFV + AZT/3TC/LPV/r + AZT/3TC/NVP + D4T/3TC/EFV + TDF/3TC/NVP. Standardized difference was calculated using Cohen d; and the difference in means or proportions was divided by the pooled standard deviation. * d > 0.10. † Data for 96 (25%) patients. ǂ Data for 184 (47%) patients. ‡ Data for 388 (100%) patients. ⁑ Data for 388 (100%) patients.

**Table 2:** Summary of the effects of the intervention on study outcomes at six months.

Outcomes	Overall (*n* = 388)	Intervention (*n* = 194)	Control (*n* = 194)	Adjusted Risk Ratio (95% CI)	*p*-Value
**Primary outcome**					
Retained on treatment	360 (93%)	183 (94%)	177 (91%)	1.03 (0.98 to 1.09)	0.24
**Secondary outcomes**					
Virally suppressed	280 (95%)	136 (93%)	144 (97%)	0.96 (0.91 to 1.01)	0.12
Labor market participation	113 (42%)	56 (40%)	57(43%)	0.93 (0.70 to 1.24)	0.63
Disclosed HIV status	278 (72%)	137(71%)	141(73%)	0.97(0.86 to 1.10)	0.65
Used Condoms for sex with unofficial partner	86 (80%)	42 (79%)	44 (82%)	0.97 (0.81 to 1.17)	0.77

CI = Confidence Interval.

**Table 3:** Factors associated with attrition from HIV treatment.

Characteristics of Study Participants	N (%)	Attrition (LTFU or Death) (%)	Hazard Ratio (95% CI)	*p*-Value	Adjusted Hazard Ratio (95% CI)	*p*-Value
Total, (*n*)%	388 (100%)	28 (100%)				
Marital status						
Married	232 (60%)	16 (57%)	1		1	
Divorced	64 (16%)	2 (7%)	0.34 (0.04–2.75)	0.313	631,925.9 (4560.265–8.76 × 10^7^)	0.000
Never married/Single	49 (13%)	6 (21%)	1.69 (0.46–6.18)	0.425	7.83 (2.02–30.34)	0.003
Widowed	43 (11%)	4 (14%)	1.39 (0.49–3.91)	0.535	2.16 (0.13–36.72)	0.595
Time to clinic (minutes)	388 (100%)	28 (100%)	0.99 (0.99–1.01)	0.982	2.86 (2.62–3.13)	0.000
Weight (Kg)	359 (93%)	20 (71%)	0.96 (0.93–0.99)	0.003	1.64 (1.55–1.74)	0.000
1st Line ART original regimen						
TDF/3TC/DTG	194 (51%)	14 (50%)	1			
TDF/3TC/EFV	182 (48%)	12 (43%)	0.81 (0.31–2.15)	0.671	0.27 (0.009–7.93)	0.449
Other ^+^	7 (1%)	2 (7%)	4.18 (0.85–20.49)	0.078	1.42 × 10^21^ (1.92 × 10^20^–1.05 × 10^22^)	0.000

^+^ Other = ABC/3TC/EFV + AZT/3TC/LPV/r + AZT/3TC/NVP + D4T/3TC/EFV + TDF/3TC/NVP. LTFU = lost to follow-up. CI = Confidence Interval.

## Data Availability

All data will be available upon request.
